# Socially Communicative Eye Contact and Gender Affect Memory

**DOI:** 10.3389/fpsyg.2019.01128

**Published:** 2019-05-22

**Authors:** Sophie N. Lanthier, Michelle Jarick, Mona J. H. Zhu, Crystal S. J. Byun, Alan Kingstone

**Affiliations:** ^1^ Brain, Attention, and Reality Laboratory, Department of Psychology, University of British Columbia, Vancouver, BC, Canada; ^2^ Atypical Perception Laboratory, Department of Psychology, MacEwan University, Edmonton, AB, Canada; ^3^ Cognition and Natural Behaviour Laboratory, Department of Psychology, University of Waterloo, Waterloo, ON, Canada

**Keywords:** gaze, eye contact, attention, memory, gender

## Abstract

Because of their value as a socially communicative cue, researchers have strived to understand how the gaze of other people influences a variety of cognitive processes. Recent work in social attention suggests that the use of images of people in laboratory studies, as a substitute for real people, may not effectively test socially communicative aspects of eye gaze. As attention affects many other cognitive processes, it is likely that social attention between real individuals could also affect other cognitive processes, such as memory. However, from previous work alone, it is unclear whether, and if so how, socially communicative eye gaze affects memory. The present studies test the assumption that socially communicative aspects of eye gaze may impact memory by manipulating the eye gaze of a live speaker in the context of a traditional recognition paradigm used frequently in the laboratory. A female (Experiment 1) or male (Experiment 2) investigator read words aloud and varied whether eye contact was, or was not, made with a participant. With both female and male investigators, eye contact improved word recognition only for female participants and hindered word recognition in male participants. When a female investigator prolonged their eye contact (Experiment 3) to provide a longer opportunity to both observe and process the investigator’s eye gaze, the results replicated the findings from Experiments 1 and 2. The findings from Experiments 1–3 suggest that females interpret and use the investigator’s eye gaze differently than males. When key aspects from the previous experiments were replicated in a noncommunicative situation (i.e., when a video of a speaker is used instead of a live speaker; Experiment 4), the memory effects observed previously in response to eye gaze were eliminated. Together, these studies suggest that it is the socially communicative aspects of eye gaze from a real person that influence memory. The findings reveal the importance of using social cues that are communicative in nature (e.g., real people) when studying the relationship between social attention and memory.

## Introduction

From the moment a child is born, she/he begins to engage with her/his mother to communicate her/his needs and to have those needs met. This is the earliest example of the importance of social interactions, and intuitively, the importance of interpreting social cues from others remains essential throughout one’s life. Our eyes are central to social interaction, as they convey a wealth of information about our emotional and mental states which people use to decode our behaviors and intentions (Emery, 2000). During a social interaction, people tend to look at other peoples’ eyes to gauge whether they are interested (Argyle et al., 1974; Ellsworth and Ross, 1975), paying attention (Kleinke et al., 1975), and what their intentions may be (Kleinke, 1986; Baron-Cohen, 1995; Emery, 2000; Shimojo et al., 2003; Ristic et al., 2005; Frischen and Tipper, 2006). Accordingly, it has been argued that one’s ability to attend to the eyes of others plays a critical role in understanding and facilitating social interaction (Kleinke et al., 1975; Cary, 1978; Campbell et al., 1990; Perrett and Emery, 1994; Emery, 2000; Vertegaal et al., 2001; Tomasello et al., 2005). On the other hand, failing to properly attend to the eyes of others has been linked to deficits in social functioning in autism spectrum disorder (see Senju and Johnson, 2009a for a review) as well as social anxiety disorder (Wieser et al., 2009; Schneier et al., 2011). Indeed, researchers have theorized that eye gaze represents a special social attentional cue (Baron-Cohen, 1995) that may be processed by dedicated neural mechanisms (such as that revealed by activity in the superior temporal sulcus, Campbell et al., 1990; Itier and Batty, 2009).

Researchers have attempted to study the eyes’ importance as a social attentional cue by using variants of classic visual attention paradigms in conjunction with socially relevant stimuli (e.g., an image of a face looking at you). In these laboratory-based tasks, such a stimulus is presented on a computer screen and a person’s eye movements – and other attentional behaviors in response to the stimulus – are recorded. Using different tasks (e.g., *free viewing*: Yarbus, 1967; Walker-Smith et al., 1977; Mojzisch et al., 2006; Birmingham et al., 2008, 2009; Kuhn et al., 2009; Schrammel et al., 2009; Foulsham et al., 2010; Foulsham and Sanderson, 2013; *attentional cueing*: Friesen and Kingstone, 1998, 2003; Driver et al., 1999; Langton and Bruce, 1999; Ristic et al., 2002; Vuilleumier, 2002; Senju and Hasegawa, 2005; Zwickel and Võ, 2010; Palanica and Itier, 2011; Wiese et al., 2012; Kuhn et al., 2014; Wykowska et al., 2014; Rensink and Kuhn, 2015; *visual search*: von Grunau and Anston, 1995; Senju et al., 2005; Doi and Ueda, 2007; Senju and Csibra, 2008; Doi et al., 2009; Palanica and Itier, 2011; and *face detection*: Macrae et al., 2002; Pageler et al., 2003; Vuilleumier et al., 2005; Conty et al., 2006, 2007; Itier et al., 2007, 2011) and a variety of stimuli (e.g., *images of faces*: Laidlaw et al., 2012; *complex scenes*: Vuilleumier et al., 2005; Birmingham et al., 2008, 2009; and *dynamic videos*: Foulsham et al., 2010; Foulsham and Sanderson, 2013; Kuhn et al., 2014; Wykowska et al., 2014), these studies show that people prefer to look at the eyes over any other feature on the face (Birmingham et al., 2008, 2009; Laidlaw et al., 2012; Levy et al., 2012) and that individuals are extremely sensitive to the signals they convey (e.g., people attend to where other people look, especially when they look at them, von Grunau and Anston, 1995; Senju et al., 2005; Vuilleumier et al., 2005; Conty et al., 2007; Doi and Ueda, 2007; Senju and Johnson, 2009b; Freeth et al., 2013).

While these laboratory tasks have made use of a variety of different social stimuli that vary in complexity and approximation to real-life social interactions, the stimuli in these tasks are seldom real people. Recently, researchers have asked similar questions about how we attend to the eyes in more natural settings, where the stimuli are live people instead of static images (Zuckerman et al., 1983; Patterson et al., 2002, 2007; Kuhn and Tatler, 2005; Tatler and Kuhn, 2007; Kuhn et al., 2008, 2016; Foulsham et al., 2011; Laidlaw et al., 2011, 2016; Risko et al., 2012; Wesselmann et al., 2012; Gallup et al., 2012a,b, 2014; Freeth et al., 2013; Wu et al., 2013, 2014; Gobel et al., 2015; Kompatsiari et al., 2018). These studies reveal that the way people respond, both behaviorally and neurologically, to a real person that they can interact with (or a robot that makes head and eye movements that resemble those of a human, Kompatsiari et al., 2018) is often different than the way they attend to an image of a person. For instance, in socially communicative settings where interactions between live people can occur (i.e., people involved in the interaction are aware that they can both send signals to and receive signals from each other; Risko et al., 2012; Gobel et al., 2015; Jarick and Kingstone, 2015; Myllyneva and Hietanen, 2015, 2016; Nasiopoulos et al., 2015; Risko and Kingstone, 2015; Conty et al., 2016; Risko et al., 2016), people will only look at one another if it is socially acceptable to do so.

While these same concerns regarding the ecological validity of attention to social stimuli should also apply to memory for social stimuli, images of people are used as stimuli in most of the work investigating how eye gaze affects memory. Some of these studies report that memory for an image of face (Hood et al., 2003; Mason et al., 2004; Smith et al., 2006) and for words (Fry and Smith, 1975; Kelley and Gorham, 1988; Macrae et al., 2002; Falck-Ytter et al., 2014) is improved when these stimuli are associated with direct gaze (though see Beattie, 1981; Conty et al., 2010; Nemeth et al., 2013). In the investigations that have used live people as stimuli, a speaker’s eye contact has been associated with improved memory for what the speaker has said (Otteson and Otteson, 1980; Sherwood, 1987; Fullwood and Doherty-Sneddon, 2006; Helminen et al., 2016). Some of the earliest studies examined how children’s academic performance could be affected by a teacher’s eye contact (Otteson and Otteson, 1980; Sherwood, 1987). For instance, Sherwood (1987) suggested that learning could be enhanced in a classroom when instructors made eye contact with members in the audience. In a more recent study, male participants remembered more details from a story told by a male storyteller who looked at them relative to a storyteller who looked away, but female participants did not (Helminen et al., 2016). The research using images and live people as stimuli seems to indicate that eye contact enhances the processing and retention of information. However, other laboratory research suggests eye contact may actually hinder performance (Beattie, 1981; Conty et al., 2010; Nemeth et al., 2013) consistent with the notion that eye contact draws attention and other cognitive resources away from the task at hand (Nemeth et al., 2013).

An important limitation of all past studies using live speakers is that researchers have generally not measured and/or systematically manipulated when a given participant actually experiences eye contact with the investigator. For example, in past studies, listeners were normally exposed to either a speaker who never made eye contact with the listener(s) in an audience or a speaker who periodically made eye contact with some undefined subset of listeners (Otteson and Otteson, 1980; Sherwood, 1987; Fullwood and Doherty-Sneddon, 2006; Helminen et al., 2016). As such, it is unclear how much eye contact a listener actually made with the speaker (if they experienced any eye contact at all). More importantly, it is also unclear whether the specific information that a listener recalled was actually the information that was presented when the speaker made eye contact, as the temporal synchrony between the speaker’s eye contact and the spoken information was not controlled (Otteson and Otteson, 1980; Sherwood, 1987; Fullwood and Doherty-Sneddon, 2006; Helminen et al., 2016). As a result, these studies cannot determine whether memory effects related to gaze reflect, for example, an enhancement or a decline from direct gaze. At best, the mixed results from the research that has used images of faces suggest that both factors may be in play. As such, it remains unclear whether mutual eye contact actually enhances or hinders memory for verbal information. What is needed is a paradigm that is controlled enough to study the effect of eye gaze, without compromising the signal that eye contact provides in a natural setting (Jarick and Kingstone, 2015; Myllyneva and Hietanen, 2015; Nasiopoulos et al., 2015; Risko and Kingstone, 2015; Conty et al., 2016; Helminen et al., 2016; Risko et al., 2016). The goal of the present work is to develop a rigorous paradigm that would avoid this limitation and enable us to investigate whether eye contact enhances or hinders memory for spoken information.

As previous laboratory work has successfully measured other gaze-related memory effects using recognition tests (e.g., gaze cuing to visual word stimuli presented on a computer screen, Fry and Smith, 1975; Kelley and Gorham, 1988; Macrae et al., 2002; Hood et al., 2003; Mason et al., 2004; Smith et al., 2006; Dodd et al., 2012; Falck-Ytter et al., 2014), the studies presented in this paper will use a variant of these classic recognition tasks. The basic methodology is as follows. In an initial study phase, a participant will be seated across from an investigator (or a video of an investigator) who reads words out loud. Critically, before the investigator reads each word s/he will either look up to make eye contact with the participant or keep gaze down at the computer screen to avoid eye contact. Afterward, the participant will perform a recognition test containing the words studied with eye contact, the words studied without eye contact, and new words. The key dependent measure will be recognition accuracy. During the study, and in order to systematically control what information is presented with eye contact, a laptop computer screen, that is only visible to the investigator, will indicate the word to be read aloud and instructions on whether or not to make eye contact with the participant on a given trial. Participants will also be instructed to make eye contact with the investigator during the experiment and to look at the investigator’s eyes if making eye contact is not possible (i.e., the investigator was looking down at the screen rather than at the participant). The investigator will monitor whether the participant makes eye contact, and participants who fail to make eye contact throughout the experiment will be excluded. In previous work, direct eye gaze has enhanced and hindered memory performance, so the effect that gaze could have in the present studies was very much an open question.

Gender has been suggested as a modulating factor in the effect of eye gaze (e.g., Helminen et al., 2016). Often eye contact helps all participants recognize a face, regardless of the their gender (Macrae et al., 2002; Hood et al., 2003; Mason et al., 2004; Smith et al., 2006). However, in some contexts, one gender will benefit from eye contact, but the other will not (e.g., Otteson and Otteson, 1980; Goodman et al., 2012; Helminen et al., 2016). Finally, researchers have observed gender differences in how attentive participants are to the eyes (Connellan et al., 2000; Lutchmaya et al., 2002) and the nonverbal signals of others (Hall, 1978; Rosenthal et al., 1979; McClure, 2000), as well as how responsive participants are to these signals (e.g., females maintain more distance between themselves and a virtual agent that makes eye contact than males; Bailenson et al., 2001; Bayliss et al., 2005). Because of this, gender should be systematically controlled since it is a factor that could influence how eye gaze affects performance. The experiments reported here use either a female (Experiments 1, 3, and 4) or a male (Experiments 2 and 4) investigator who looks at male and female participants. This experimental setup has the added benefit of permitting an examination of whether gender will influence any observed eye gaze-induced memory effects.

## Experiment 1

It is currently unclear whether socially communicative eye contact helps or hinders memory. To determine this, we manipulated whether an investigator reading words aloud made eye contact with a participant or not and determined how this manipulation affected participants’ word recognition. If the investigator’s eye contact is helpful when a participant encodes information, then recognition performance would be best for words spoken while the investigator made eye contact. Alternatively, if the investigator’s eye contact interferes with encoding, recognition performance would be worse for words spoken while the investigator made eye contact with the participant. Since the gender of both participants’ and the gaze cue (e.g., investigator) have been reported to modulate the effect of gaze on memory, this factor was systematically manipulated across the studies reported in this paper. In Experiment 1, the investigator was female.

### Method

#### Participants

Eighty-four undergraduate students from the University of British Columbia (42 males, 42 females) received course credit for participating. All reported speaking English as their first language. All had normal or corrected to normal vision and were naive about the purpose of the experiment. All participants gave informed consent before participating and all associated methods were approved by the University of British Columbia’s Research Ethics Board [Towards a More Natural Approach to Attention Research 1-200, certificate #H10-00527, & Research in Cognitive Ethology, #H04-80767].

#### Design

A 2 (Investigator gaze: eye contact and no eye contact) by 2 (Participant gender: male and female) mixed design was used, where investigator gaze was manipulated within participant and participant gender was a between-participant variable.

#### Apparatus

E-Prime 2.0^1^ controlled the timing and presentation of stimuli read aloud by the investigator to the participant and logged response accuracy and response times (RTs) in the recognition test. The stimuli were presented on a 17-in. monitor with a 1920 × 1,080 pixel resolution.

#### Stimuli

The stimulus pool consisted of the 120 words from Macdonald and Macleod (1998). The words were nouns 5–10 letters long, with frequencies greater than 30 per million (Thorndike and Lorge, 1944). From the 120 words, 3 lists containing 40 words each were randomly generated. For a given participant, two lists were selected for study; one list was presented with eye contact and the other list without. The third list was reserved for a recognition test. List selection was counterbalanced across participants such that each word was presented in each of the different conditions (i.e., with eye contact, without eye contact, new words for recognition) an even number of times across participants.

#### Procedure

Participants heard words for a later memory test. Participants were not informed that they would complete a memory test after hearing the words. During the initial encoding phase, participants were seated ~40 in. across from a female investigator who read aloud words individually. Critically, while the investigator read the words, she either looked up to make eye contact briefly (less than a second) with the participant or kept gaze down at the computer screen to avoid eye contact. Eighty words in total were read aloud in random order to the participants, half of which were presented with eye contact and the other half without.

A laptop screen (only visible to the investigator) indicated when a word was to be read aloud and provided instructions on whether or not to make eye contact with the participant on a given trial. To begin each trial, a blank screen appeared for 1,500 ms. Next, the instruction to look up at the participant or look down at the laptop was presented to the investigator. After 1,000 ms, a word also appeared and remained on screen for 3,000 ms. As soon as the word appeared on screen, the investigator would then look as instructed either toward the participant or down at the computer screen while she read the word aloud. On trials where the investigator made eye contact, the investigator would look back down at the computer screen as she finished saying the word (~1 s). Next, a blank white screen would appear for 500 ms to alert the investigator of the end of the trial. The words and eye contact instructions were randomly intermixed. Since neither the investigator nor participant had knowledge of the trial sequence, this would prevent any systematic change in a participant’s eye gaze throughout the experiment. The investigators (who were authors MZ and CB) were trained to maintain a neutral facial expression and a consistent tone of voice irrespective of the gaze condition, though some natural non-systematic variation within and across participants was expected. The investigators memorized the instruction script for each phase, followed the instruction prompt on the computer screen (i.e., “look at participant or look at screen”), and subsequently read the words to the participant. Additionally, investigators rehearsed before testing to help maintain consistency.

**Figure 1:**
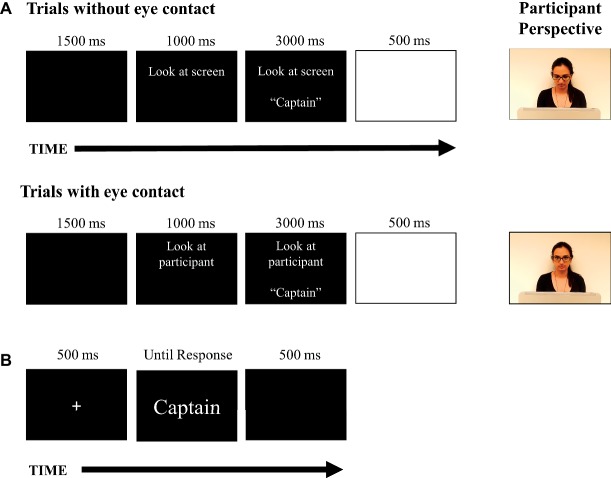
The depiction of the experimental setup and procedure used in Experiment 1. **(A)** The instructional sequence that was visible to the investigator for different trials during the encoding phase. When instructed, the investigator would lift her eyes to make eye contact with the participant as the word appeared on screen and was read aloud. The investigator is depicted from the participant’s perspective on each trial type. **(B)** The trial sequence that was presented to the participants during the recognition phase of the experiment.

To further ensure that eye contact between the investigator and participant was controlled, participants were instructed to make eye contact with the investigator during the experiment, and if making eye contact was not possible (i.e., the investigator was looking down at the screen) to look at the investigator’s face. This way, eye contact could be made easily when the investigator looked up at the participant. Thus, eye contact was monitored by the investigator on a trial-by-trial basis. In instances when a participant failed to make eye contact with the investigator consistently (e.g., on one or more trials), the participant was excluded. Any failures to make eye contact were extremely rare, and of the 2 (out of 84) participants who were excluded, there was no ambiguity, i.e., they consistently failed to make eye contact. It is important to note that that people are very sensitive at judging where people are looking and when eye contact is being made, even when judging that behavior *via* a static image or video (e.g., Anderson et al., 2011). The instructional sequence visible to the investigator during the encoding phase is presented in Figure 1A.

Once the encoding phase was complete, the investigator would open a recognition test on the laptop and turn the laptop to face the participant. After reading the recognition test instructions to the participant, the investigator would monitor the participant’s performance as they completed four practice trials (which were excluded from the analysis). Next, the investigator left the participant alone in the room to complete the recognition test. The recognition test contained the words studied with eye contact, the words studied without eye contact, and 40 new words. The test words appeared on a computer screen in white font against a black background and were presented in random order. A fixation cross was presented for 500 ms before each word. When a word appeared, the subjects were instructed to make a “new” or “old” response for each test word by pressing buttons labeled “New” and “Old” on the keyboard. There was a 500-ms blank interval before each word appeared on screen, and the word offset with the subject’s key response. The response accuracy and response times were recorded. The trial sequence used during the recognition phase trial is presented in Figure 1B. Once the recognition task was complete, the participant remained seated until the investigator came back to the room.

### Results

A two-way mixed ANOVA was conducted on response time (RT) and response accuracy (percentage correct), with investigator gaze (two levels: with eye contact and without eye contact) as the within-participant factor and participant gender (two levels: male and female) as the between-participant factor.

#### Response Time

**Figure 2:**
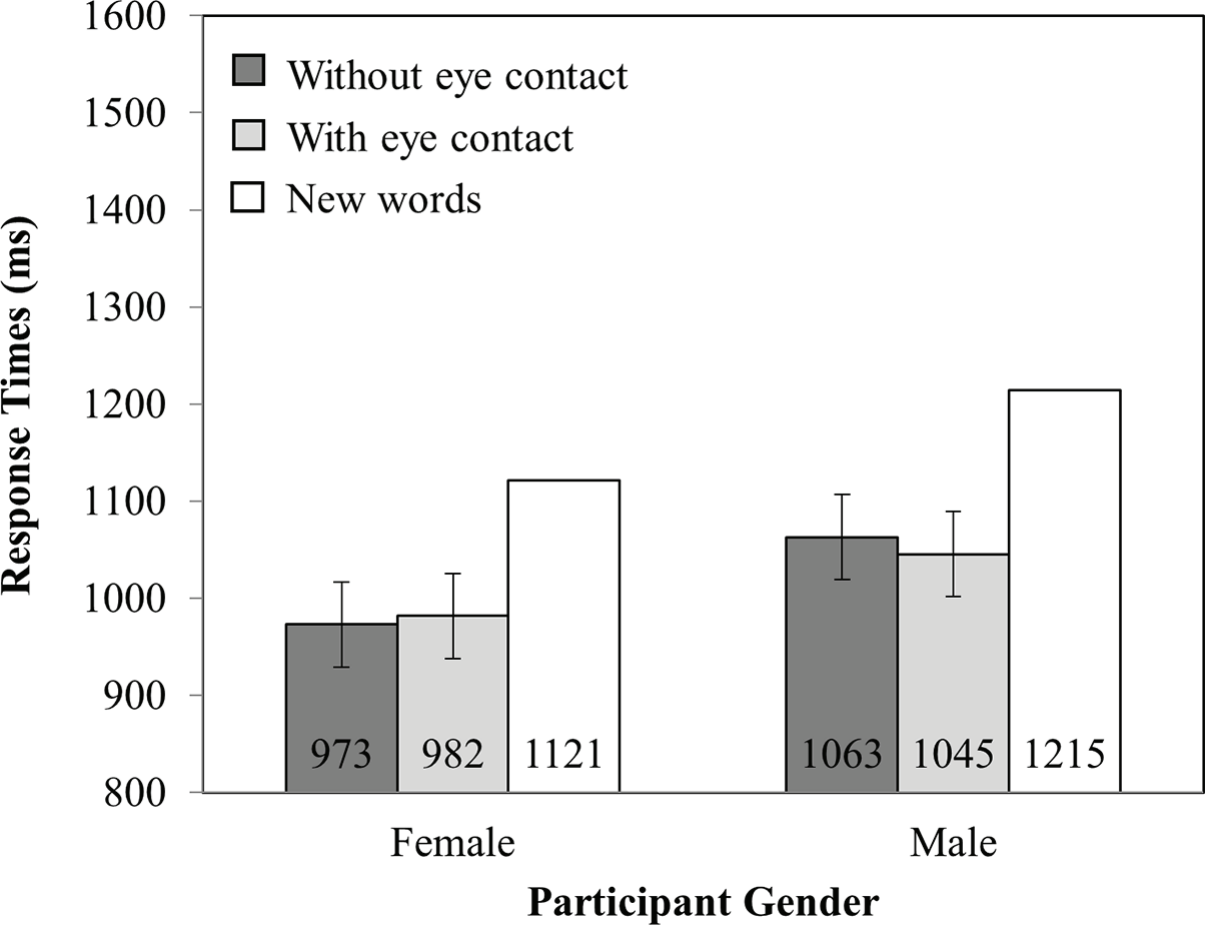
RT as a function of Participant Gender (Female versus Male) and Investigator gaze (With eye contact versus Without eye contact). Note that new words have been plotted in this figure as a reference point but were not included in the analysis. Error bars represent the 95% confidence interval as defined by Masson and Loftus (2003).

Mean RTs are presented in Figure 2. There were no main effects of investigator gaze (*F*_(1,82)_ = 0.04, MSE = 20,386.56, *p* = 0.84) or participant gender (*F*_(1,82)_ = 1.08, MSE = 229,174.88, *p* = 0.30). Nor was there an interaction between investigator gaze and participant gender (*F*_(1,82)_ = 0.36, MSE = 20,386.56, *p* = 0.55).

#### Percentage Correct

**Figure 3:**
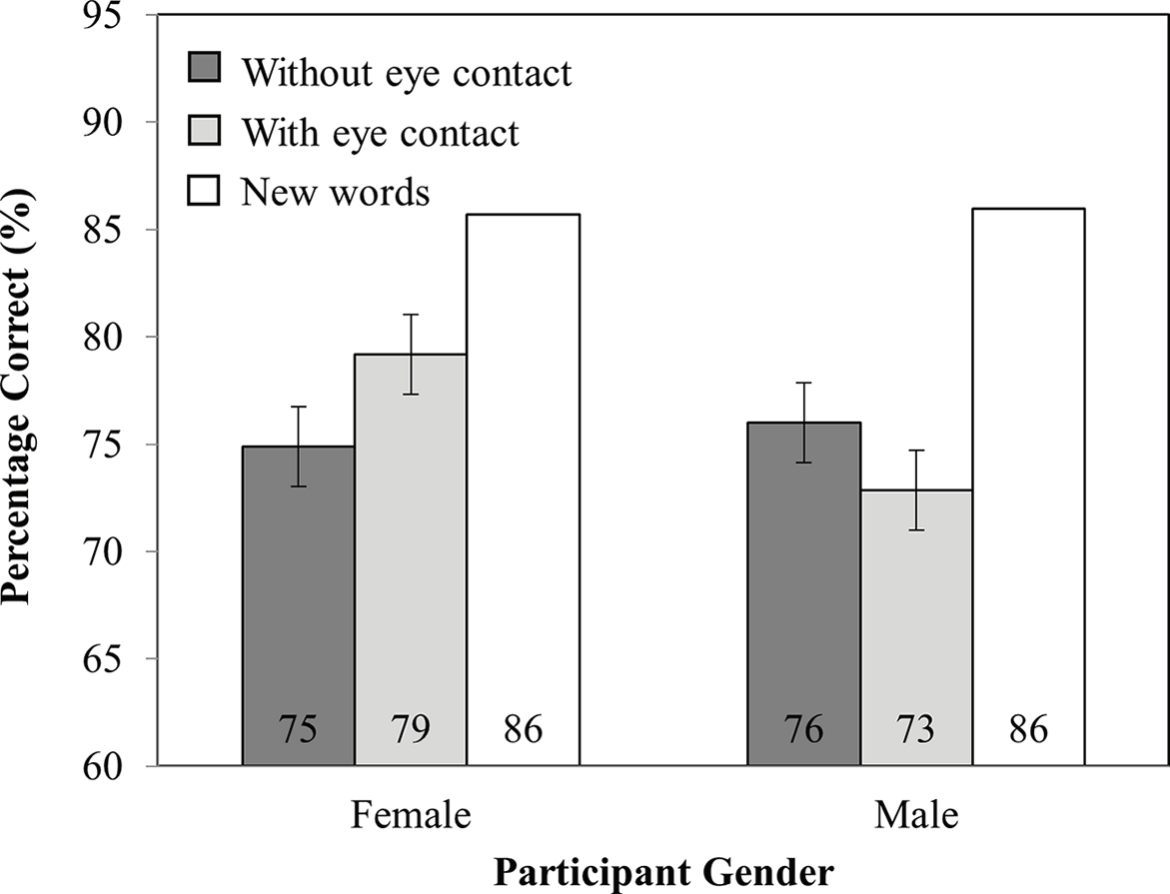
Percentage correct as a function of Participant Gender (Female versus Male) and Investigator gaze (With eye contact versus Without eye contact). Note that new words have been plotted in this figure as a reference point but were not included in the analysis. Error bars represent the 95% confidence interval as defined by Masson and Loftus (2003).

Analysis of the accuracy data (see Figure 3) revealed no main effect of investigator gaze (*F*_(1,82)_ = 0.37, MSE = 36.69, *p* = 0.55) or participant gender (*F*_(1,82)_ = 0.88, MSE = 321.28, *p* = 0.35). Critically, there was an interaction between investigator gaze and participant gender (*F*_(1,82)_ = 15.84, MSE = 36.69, *p* < 0.001), such that female participants recognized *more* words that were spoken while the investigator made eye contact (79%) than when they did not (75%; *t*_(41)_ = 3.27, SEM = 1.31, *p* < 0.005). However, male participants recognized *fewer* words read while the investigator made eye contact (73%) than when they did not (76%; *t*_(41)_ = 2.37, SEM = 1.33, *p* < 0.05).

### Discussion

The results from this initial experiment demonstrate that memory is improved for words that were encoded with eye contact, but this effect was only observed in female participants. These findings suggest that eye contact has differential effects on memory for verbal information in males and females. Given that females are more attentive and responsive to nonverbal behavior than males (Hall, 1978; Connellan et al., 2000; Bailenson et al., 2001; Lutchmaya et al., 2002; Bayliss et al., 2005; Yee et al., 2007; Marschner et al., 2015), it is possible that, in the context of the present study, females dedicated more attention to information delivered during eye contact than males, which may have resulted in deeper processing and better retention of words presented with than without eye contact (Craik and Tulving, 1975).

Alternatively, it could be the case that making eye contact with the opposite sex produces higher levels of arousal compared to making eye contact with the same sex (Argyle and Dean, 1965; Donovan and Leavitt, 1980). Research has demonstrated that eye contact elevates physiological arousal (Kleinke and Pohlen, 1971; Nichols and Champness, 1971; Gale et al., 1978; Wieser et al., 2009; Helminen et al., 2011, 2016), and that high levels of arousal can interfere with performance on similar tasks (Jelicic et al., 2004; Smeets et al., 2007). While it is possible that eye contact holds one’s attention by increasing arousal (i.e., affective arousal theory; Kelley and Gorham, 1988; Senju and Johnson, 2009b; Mather and Sutherland, 2011), eye contact between genders could produce excess arousal and anxiety, and thus, interfere with memory. Accordingly, male participants could have experienced more arousal than the female participants while making eye contact with the female investigator and were more distracted while words spoken with eye contact as a result (Nemeth et al., 2013). Since both the task and processing the eye contact are competing for cognitive resources, performance on the cognitive task suffers. We examined these possibilities in Experiment 2.

## Experiment 2

In the previous experiment, only female participants benefited from the investigator’s gaze. This finding could be attributed to the female investigator’s eye contact distracting male participants from processing what is said (Nemeth et al., 2013). It could be the case that female participants were simply more attentive to the female investigator’s eye contact than males irrespective of the investigator’s gender (Hall, 1978; Connellan et al., 2000; Bailenson et al., 2001; Lutchmaya et al., 2002; Bayliss et al., 2005; Yee et al., 2007; Marschner et al., 2015). We seek to distinguish between these possibilities in Experiment 2 by using a male investigator. If males now benefit from eye contact (and females are possibly hindered by eye contact), this would support the idea that the investigator’s gender contributes to the memory effect *vis-à-vis* its relation to the participant. However, if the results replicate Experiment 1, then a participant’s gender is a contributing factor to how eye gaze influences memory, a finding that would also be consistent with the notion that females interpret nonverbal social cues differently than males.

### Method

#### Participants

Eighty-four undergraduate students from the University of British Columbia (42 males, 42 females) who had not previously participated in Experiment 1 received course credit for participating. All had normal or corrected to normal vision and were naive about the purpose of the experiment.

#### Design, Apparatus, Stimuli, and Procedure

The design, apparatus, stimuli, and procedure were identical to those used in the previous study, with the exception that now a male investigator read the words aloud to the participants instead of a female investigator.

### Results

Data analysis followed the same procedure that was used in Experiment 1.

#### Response Time

**Figure 4:**
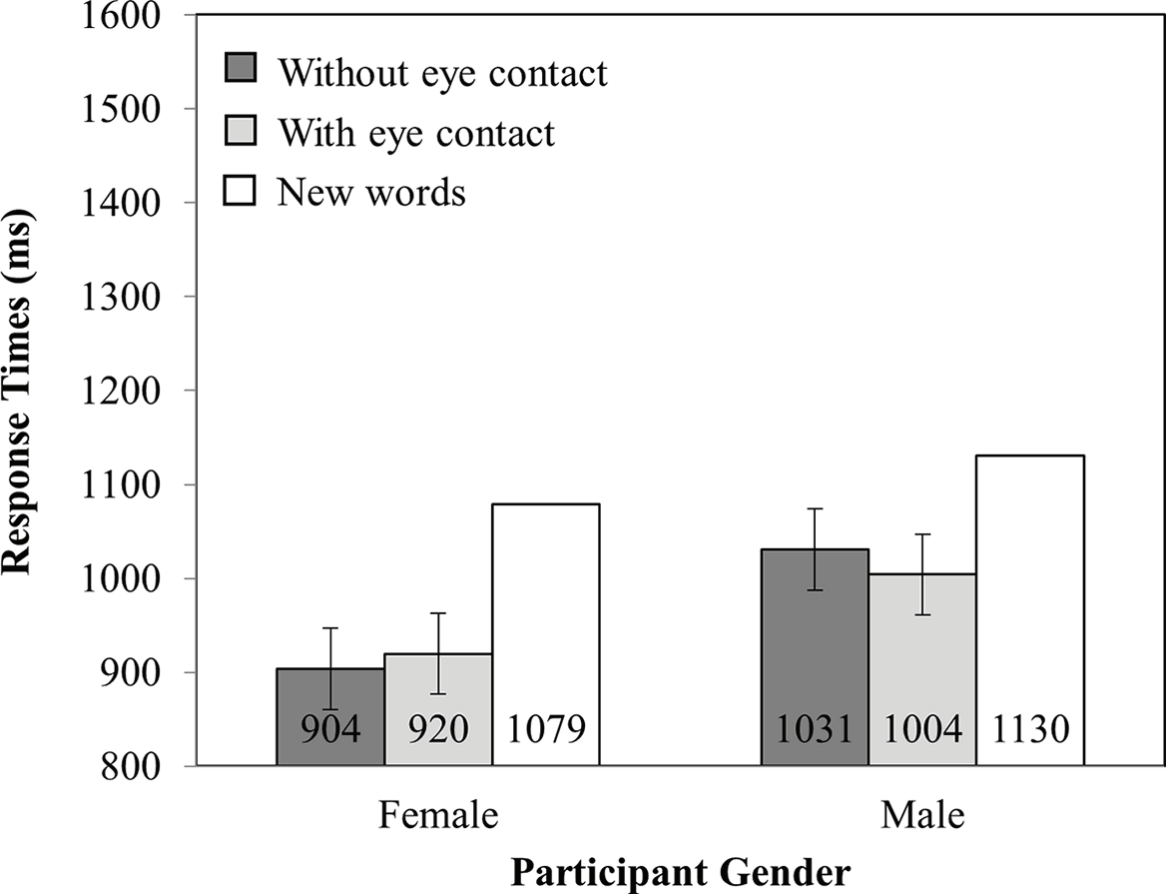
RT as a function of Participant Gender (Female versus Male) and Investigator gaze (With eye contact versus Without eye contact). Note that new words have been plotted in this figure as a reference point, but were not included in the analysis. Error bars represent the 95% confidence interval as defined by Masson and Loftus (2003).

Mean RTs are presented in Figure 4. There was a marginally significant main effect of participant gender (*F*_(1,82)_ = 3.35, MSE = 140,584.08, *p* = 0.07), such that females (967 ms) were faster to respond than males (1,055 ms). No other main effects or interaction were significant (all other *F*’s < 1).

#### Percentage Correct

**Figure 5:**
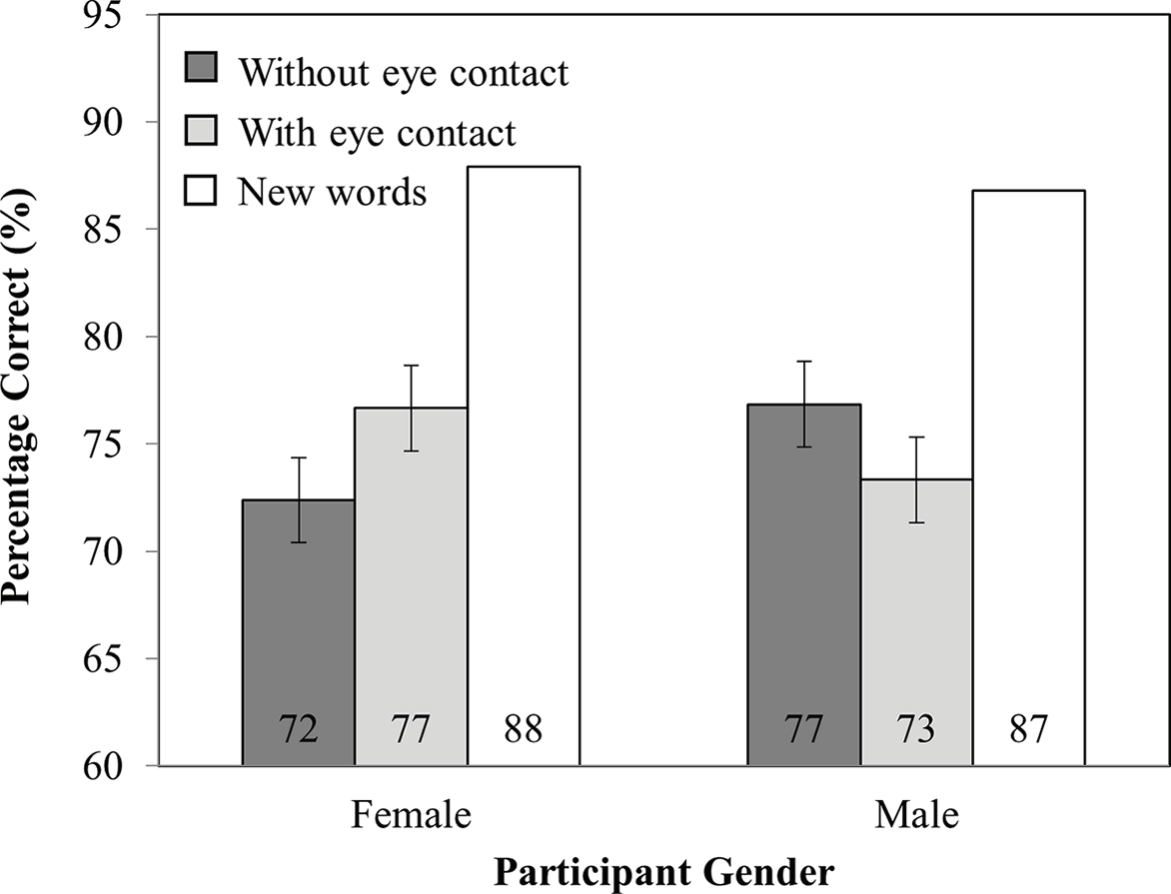
Percentage correct as a function of Participant Gender (Female versus Male) and Investigator gaze (With eye contact versus Without eye contact). Note that new words have been plotted in this figure as a reference point, but were not included in the analysis. Error bars represent the 95% confidence interval as defined by Masson and Loftus (2003).

Analysis of the accuracy data (see Figure 5) revealed no main effect of investigator gaze (*F*_(1,82)_ = 0.15, MSE = 41.95, *p* = 0.70) or participant gender (*F*_(1,82)_ = 0.03, MSE = 463.44, *p* = 0.87). Critically, there was an interaction between investigator gaze and participant gender (*F*_(1,82)_ = 15.22, MSE = 41.95, *p* < 0.001), such that female participants recognized *more* words that were spoken while the investigator made eye contact (77%) than when they did not (72%; *t*_(41)_ = 3.68, SEM = 1.17, *p* < 0.001). However, male participants recognized *fewer* words read while the investigator made eye contact (73%) than when they did not (77%; *t*_(41)_ = 2.16, SEM = 1.62, *p* < 0.05).

### Discussion

Results from Experiment 2 replicate the findings reported in Experiment 1, wherein females benefited from eye contact on recognition tests and males did not. Furthermore, the present experiment rules out a same-gender explanation of Experiment 1, where memory was improved only when making eye contact with a person of the same gender (i.e., female investigator and female participants). Here, female participants showed a memory benefit from eye contact with a male investigator as well. These results are consistent with the notion that females are generally more attentive to gaze cues than males (Hall, 1978; Connellan et al., 2000; Bailenson et al., 2001; Lutchmaya et al., 2002; Bayliss et al., 2005; Yee et al., 2007; Marschner et al., 2015). It is possible that female participants decode the various signals that could be embedded within the investigator’s eye contact (e.g., a signal to pay attention, a signal that the investigator is watching you, information about the investigator’s mental state, etc.) more readily than males. Thus, females quickly interpret this eye contact as a signal to pay attention, and dedicate more cognitive resources to words presented while the investigator looks at them (e.g., Otteson and Otteson, 1980; Sherwood, 1987). On the other hand, males may find interpreting the investigator’s eye contact distracting since it is uninformative to the task, or perhaps they require more cognitive resources than females to process the different social signals. As a result, their performance on the task at hand is impaired.

## Experiment 3

In the two previous experiments, the eye contact that was initiated by the investigator was quite brief (i.e., a quick glance (less than 1 s) up at the participant as the investigator said the word aloud). Although this brief glance may have provided enough time for females to decode the eye contact, it is possible that male participants needed longer periods of eye contact in order to decode this social cue. Indeed, in more natural settings, people tend to engage in eye contact with others between 1.7 and 3.6 s (Argyle and Dean, 1965; Helminen et al., 2011). To determine whether more eye contact can help males decode social cues more effectively, in the present study, the investigator made eye contact for a longer period of time (approximately 3 s). In order to maximize any effect of prolonged eye contact on the male participant’s arousal, whether it is beneficial arousal or distracting arousal, we used a female investigator. If prolonged durations enable men to use eye contact as a social cue to enhance verbal information processing, then the present study should reduce or eliminate the effects of participant gender on word recognition.

### Method

#### Participants

Eighty-four undergraduate students from the University of British Columbia (42 males, 42 females) who had not previously participated in Experiment 1 or 2 received course credit for participating. All had normal or corrected to normal vision and were naive about the purpose of the experiment.

#### Design, Apparatus, Stimuli, and Procedure

The design, apparatus, stimuli, and procedure were identical to those used in Experiments 1 and 2, with the exception that the female investigator made *prolonged eye contact* when instructed to look at the participant instead of brief eye contact to further accentuate any effect of eye gaze. As in the previous experiments, a laptop that only the investigator could see provided the same instructions on what word to say and whether to make eye contact or not as the word was spoken. On trials where the investigator made eye contact with the participant, the investigator lifted her eyes and read the word when the laptop displayed the word and made continuous eye contact with the participant until the screen flashed white after 3,000 ms, at which point the investigator would return her gaze down to the computer screen.

### Results

Data analysis followed the same procedure that was used in Experiments 1 and 2.

#### Response Time

**Figure 6:**
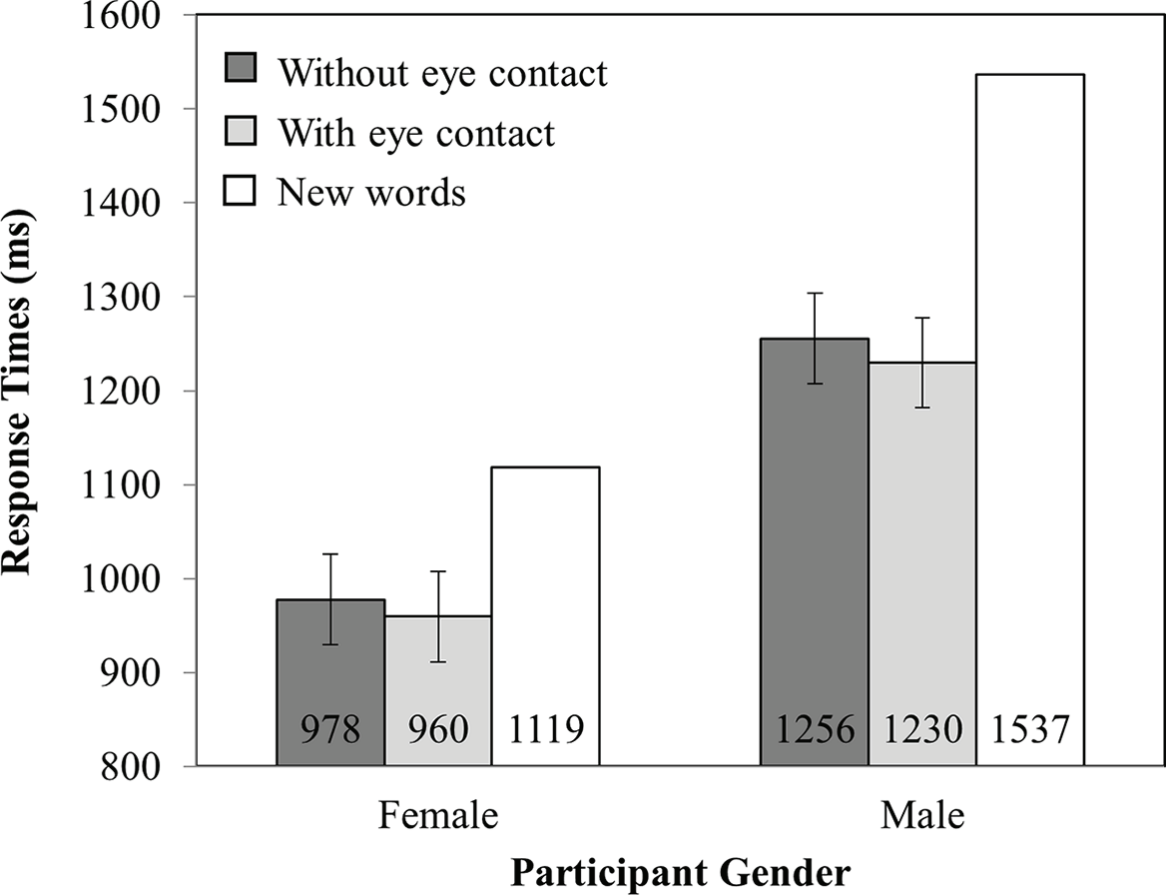
RT as a function of Participant Gender (Female versus Male) and Investigator gaze (With eye contact versus Without eye contact). Note that new words have been plotted in this figure as a reference point, but were not included in the analysis. Error bars represent the 95% confidence interval as defined by Masson and Loftus (2003).

Mean RTs are presented in Figure 6. There was a main effect of participant gender (*F*_(1,82)_ = 15.71, MSE = 200,664.95, *p* < 0.001), such that females (1,019 ms) were faster to respond than males (1,341 ms). No other main effects or interactions were significant (all other *F’s* < 1).

#### Percentage Correct

**Figure 7:**
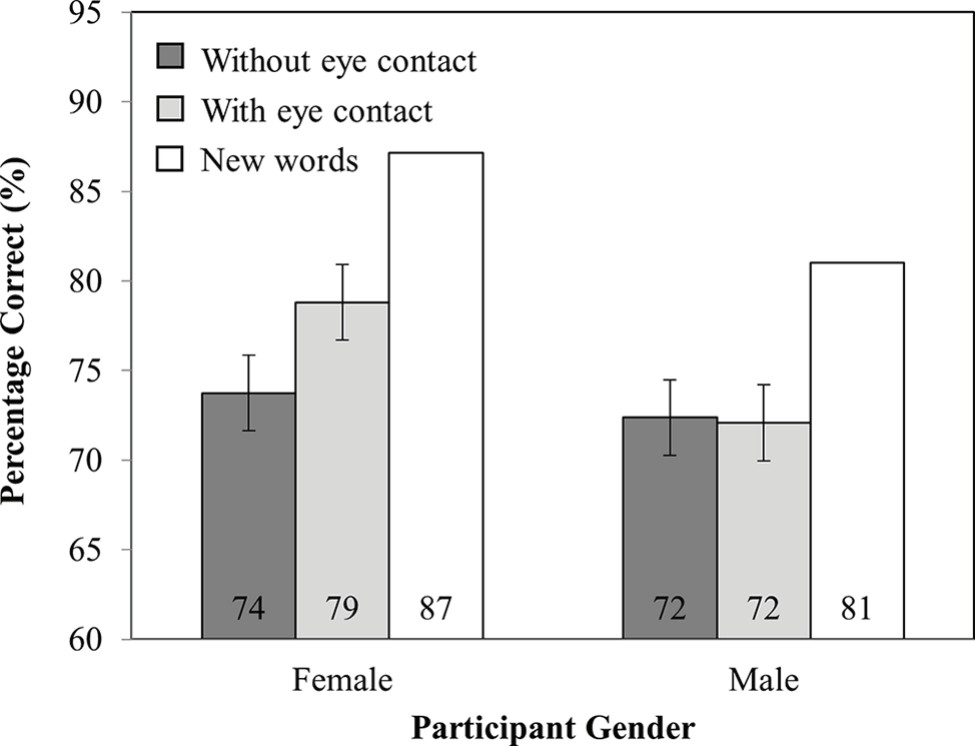
Percentage correct as a function of Participant Gender (Female versus Male) and Investigator gaze (With eye contact versus Without eye contact). Note that new words have been plotted in this figure as a reference point, but were not included in the analysis. Error bars represent the 95% confidence interval as defined by Masson and Loftus (2003).

Analysis of the accuracy data (see Figure 7) revealed a main effect of investigator gaze (*F*_(1,82)_ = 5.03, MSE = 47.31, *p* < 0.05), such that words presented with investigator eye contact (75%) were accurately recognized more than those presented without (73%). There was no main effect of participant gender (*F*_(1,82)_ = 1.77, MSE = 389.94, *p* = 0.19). Critically, there was an interaction between investigator gaze and participant gender (*F*_(1,82)_ = 6.37, MSE = 47.31, *p* < 0.02), such that female participants recognized *more* words that were spoken while the investigator made eye contact (79%) than when they did not (74%; *t*_(41)_ = 3.45, SEM = 1.47, *p* < 0.001). However, male participants were no more likely to recognize words read with (72%) or without investigator eye contact (72%; *t*_(41)_ = 0.19, SEM = 1.53, *p* = 0.54).

#### Comparison Between Experiments 1, 2, and 3

An additional follow-up analysis comparing all three experiments was run to reveal any differences (or similarities) in the effects eye contact had on memory in each experiment. A three-way mixed ANOVA was conducted on mean response accuracy with investigator gaze (two levels: with eye contact and without eye contact) as the within-participant factor and experiment (three levels: female investigator with brief glance, male investigator with brief glance, and female investigator with prolonged gaze) and participant gender (two levels: male and female) as between-participant factors. The analysis of the response accuracy data revealed a marginal main effect of investigator gaze (*F*_(1,246)_ = 3.67, MSE = 42.00, *p* = 0.06), such that participants recognized more words that the investigator said while making eye contact than when they did not. Critically, this was qualified by a reliable interaction between investigator gaze and participant gender (*F*_(1,246)_ = 35.16, MSE = 42.00, *p* < 0.001), such that female participants recognized *more* words that were spoken while the investigator made eye contact (78%) than when they did not (74%; *t*_(125)_ = 6.00, SEM = 0.76, *p* < 0.001). However, male participants recognized *fewer* words read while the investigator made eye contact (73%) than when they did not (75%; *t*_(125)_ = 2.67, SEM = 0.87, *p* < 0.01). No other main effects or interactions were significant (all *F*’s < 1.27). Note that the lack of a three-way interaction (*p* = 0.65) is supported by a Bayes factor estimated using Monte Carlo sampling *via* the BayesFactor package in R (Morey and Rouder, 2018). Specifically, the Bayes factor was 0.125:1 when comparing a model with the three-way interaction to a model without (i.e., only two-way interactions and main effects). In other words, there is about eight times more evidence against including a three-way interaction in the model.

### Discussion

The results of Experiment 3 replicated the finding in both Experiments 1 and 2 that females recognized more words and were more sensitive to words presented with eye contact than without eye contact. Unlike the previous studies, male participants’ recognition performance was not significantly different in the eye contact and no eye contact conditions. Further, the follow-up analysis comparing all three experiments revealed that the length of eye contact did not modify the effect eye contact had on memory in female participants.

The failure to observe any interaction between Experiment and Investigator gaze or Experiment, Investigator gaze, and Participant gender suggests that exaggerating eye contact does not help the males encode the nonverbal eye contact cues, nor did it enhance the eye contact benefit observed in female participants (as compared to female participants in Experiments 1 and 2)^2^. These results also suggest that the memory benefits that arise from eye contact in Experiments 1–3 are unlikely to be mediated by an arousal response, since prolonging the eye contact in the present experiment would have, if anything, increased arousal, which could modify how eye contact affects performance^3^. Instead, we suggest that these results are more consistent with the idea that eye contact provides a signal to pay attention, and that interpreting this social signal is responsible for enhancing information processing in the previous studies. If anything, it seems that lengthening the investigator’s eye contact reduced the interference eye contact caused the males in Experiments 1 and 2. By providing the signal longer, males may have had enough resources to both process the eye contact and perform the memory task without these two tasks competing for cognitive resources. Since they did not perform better when the investigator made eye contact than when they did not, it seems that this cue may not have had social relevance for the males.

The interpretations discussed here all assume that the investigator’s eye gaze is being interpreted (at least by female participants) as a socially communicative cue. While the live interaction between the investigator and the participant ensures that social communication can occur, it is also possible that a nonsocial cue associated with the investigator’s eye gaze could also be driving the reported effects. Before concluding that socially communicative aspects of gaze produce these effects, it is important to exclude a nonsocial interpretation that could possibly account for the facilitatory effect of gaze. This will be addressed in Experiment 4 where the social communicative aspects of eye gaze will be dissociated from the purely perceptual cues by using a video of the investigator instead of a live investigator.

## Experiment 4

The previous studies demonstrated that females benefited from an investigator’s gaze on a subsequent memory test, whereas males did not. These gender-specific memory effects could be driven by a socially communicative cue that is embedded in the investigator’s eye contact (i.e., when someone looks at you, it is a signal to pay attention). According to this idea, females might have been sensitive to the social cue embedded in the investigator’s eye contact, and could have used it to facilitate their performance on the recognition test. However, male participants might have failed to interpret and apply the investigator’s eye contact as a signal to pay attention, and as a result their performance at test could have been hindered by the investigator’s eye gaze.

A different possibility altogether is that there was nothing socially communicative about the investigator’s gaze that drove the memory effects observed in the previous studies. For example, these effects could have arisen by observing the investigators shifting their gaze up from the computer monitor. In the previous studies, the investigators either kept their eyes on the computer screen while they read a word, or they lifted them to make eye contact just before saying a word. Observing just the movement of the eyes up from the computer screen could be an indicator that a word is about to be spoken, much in the same way the onset of a flashing light at a crosswalk indicates that one should pay attention for pedestrians. There is nothing inherently “social” about either of these cues, but they both serve the purpose of a warning cue that informs a participant to increase attention to an upcoming stimulus (i.e., a word or a pedestrian in the latter case). In fact, a variety of perceptual cues (i.e., arrows, flashes in the periphery, etc.) are known to generate changes in attention (e.g., Posner, 1980; Friesen et al., 2004; Bayliss et al., 2005; Hietanen et al., 2006; Ristic et al., 2007; Mulckhuyse and Theeuwes, 2010; Shin et al., 2011; Hayward and Ristic, 2015). Given that both perceptual and socially communicative cues were embedded in the live investigator’s eye contact in the previous experiments, it is unclear which cue was actually driving the memory effects observed in the previous experiments.

The aim of Experiment 4 is to clarify whether socially communicative cues are responsible for the eye gaze-related effects observed in the previous experiments. One way to isolate the social aspects of eye gaze from the perceptual ones is to have observers watch a video of the investigator instead of interacting with a live investigator. Numerous studies have demonstrated that people respond differently, both behaviorally and neurologically, when looking at the eye gaze of people presented in images versus actual, physically present people (Hietanen et al., 2008; Itier and Batty, 2009; Teufel et al., 2010; Laidlaw et al., 2011; Pönkänen et al., 2011a,b; Risko et al., 2012, 2016; Schilbach et al., 2013; Schilbach, 2015). Furthermore, the eye gaze and gestures of people depicted in images and videos have less influence on the communication (Heath and Luff, 1993; Gullberg and Holmqvist, 2006) and attention (Varao-Sousa and Kingstone, 2015; Wammes and Smilek, 2017) of an observer than they typically would during an encounter with a live person. Presumably, this is because the people depicted in the images and videos cannot see the observer and therefore their gaze behavior is not actively communicating with the observer, and *vice versa* (De Jaegher et al., 2010; Schilbach, 2010; Risko et al., 2012).

By using a video recording of the investigator in the present study, the socially interactive context that was produced by using a live investigator in Experiments 1, 2, and 3 is removed. If the previous findings are replicated, it would suggest that perceptual cues derived from the eye gaze of someone in a video are enough to generate the memory benefits and deficits associated with eye contact, and that a socially communicative context is not required to generate these memory effects. However, eliminating eye-gaze related memory effects would be evidence for the idea that perceptual cues are not driving these previously observed effects. Instead, it would suggest that the socially communicative eye gaze from an individual that an observer could potentially interact with is required to produce these memory effects.

### Method

#### Participants

To examine investigator gender as a factor in one experiment rather than in two separate experiments, as was the case in Experiments 1 and 2, the sample size was doubled to 168 undergraduate students from the University of British Columbia (84 males, 84 females) who had not participated in any of the previous experiments. All received course credit for participating and had normal or corrected to normal vision and were naive about the purpose of the experiment.

#### Design

A 2 (Investigator gaze: eye contact and no eye contact) by 2 (Participant gender: male and female) by 2 (Investigator gender: male and female) mixed design was used, where investigator gaze was manipulated within participant and participant gender and investigator gender were a between-participant variables.

#### Apparatus and Stimuli

The apparatus and stimuli were identical to those reported in the Experiments 1 and 2; however, the participants now watched a video of the investigator from either Experiment 1 (female investigator) or Experiment 2 (male investigator). The videos shown to each participant were recorded by a camera that was placed in front of the investigator, on a tripod that was adjusted so that the camera was positioned at the investigator’s eye level. This position was chosen to simulate the distance, height, and eye level of a participant who would have sat across from the live investigator in the previous experiments. A confederate also sat directly behind the camera and looked at the investigator’s eyes. The intention was to simulate the same live interaction to ensure that any variation in expressiveness due to a live context (Experiments 1–3) was also generated when the investigator was videotaped. During the recordings, the investigator read the words aloud as in the previous experiments, i.e., when prompted by the laptop, the investigator either looked toward the computer screen or, to simulate eye contact for the viewer, briefly toward the camera lens. A total of six different videos were made to ensure that across participants, each word would appear in each condition evenly.

Videos were presented full screen at the recorded resolution (1,920 × 1,080 pixels), on a 17-in. monitor. Participants were seated approximately 60 cm from the screen. Sound from the videos was also played through speakers built into the computer.

#### Procedure

The procedure was identical to those used in Experiments 1 and 2, with the exception that a participant was first assigned to watch a video of either a male or female investigator saying the words out loud. Participants were also instructed to look at the investigators’ eyes throughout the experiment. Based on a wealth of past work indicating that there is a preferential bias to look at the eyes of people when they are shown in photos or in videos (Laidlaw et al., 2011; Risko et al., 2012, 2016), we expected participants to readily comply with the instructions. Their performance during practice and self-report after testing support this prediction.

### Results

A three-way mixed ANOVA was conducted on response time (RT) and response accuracy, with investigator gaze (two levels: eye contact and no eye contact) as the within-participant factor and participant gender (two levels: male and female) and investigator gender (two levels: male and female) as the between-participant factors.

#### Response Time

**Figure 8:**
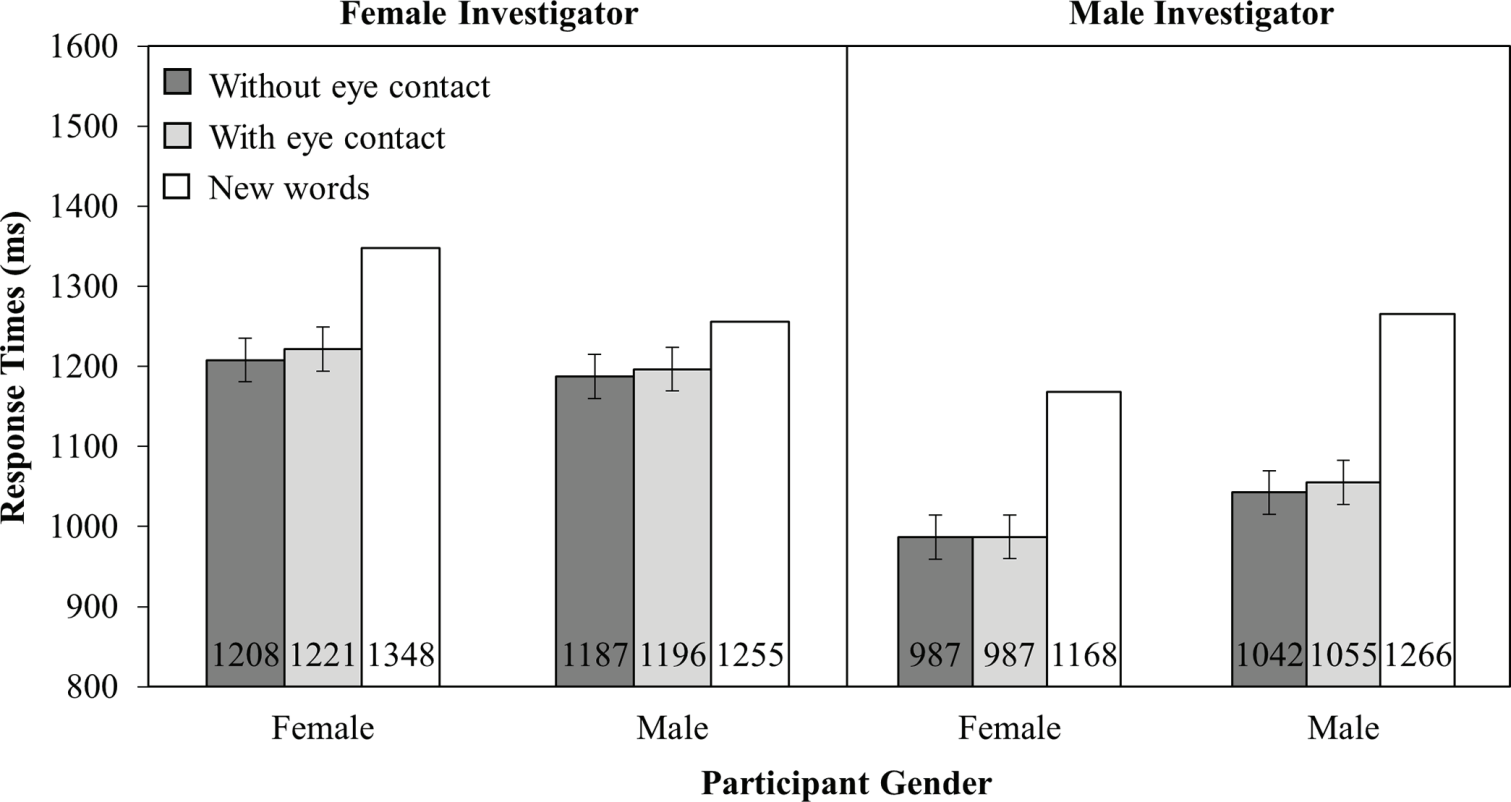
RT as a function of Investigator Gender (Female versus Male), Participant Gender (Female versus Male), and Investigator gaze (With eye contact versus Without eye contact). Note that new words have been plotted in this figure as a reference point, but were not included in the analysis. Error bars represent the 95% confidence interval as defined by Masson and Loftus (2003).

Mean RTs are presented in Figure 8. There was a main effect of investigator gender (*F*_(1,166)_ = 13.47, MSE = 214,475.60, *p* < 0.001), such that participants were faster to respond with the male investigator (1,018 ms) than the female investigator (1,203 ms). No other main effects or interactions were significant (all other *F’s* < 1).

#### Percentage Correct

**Figure 9:**
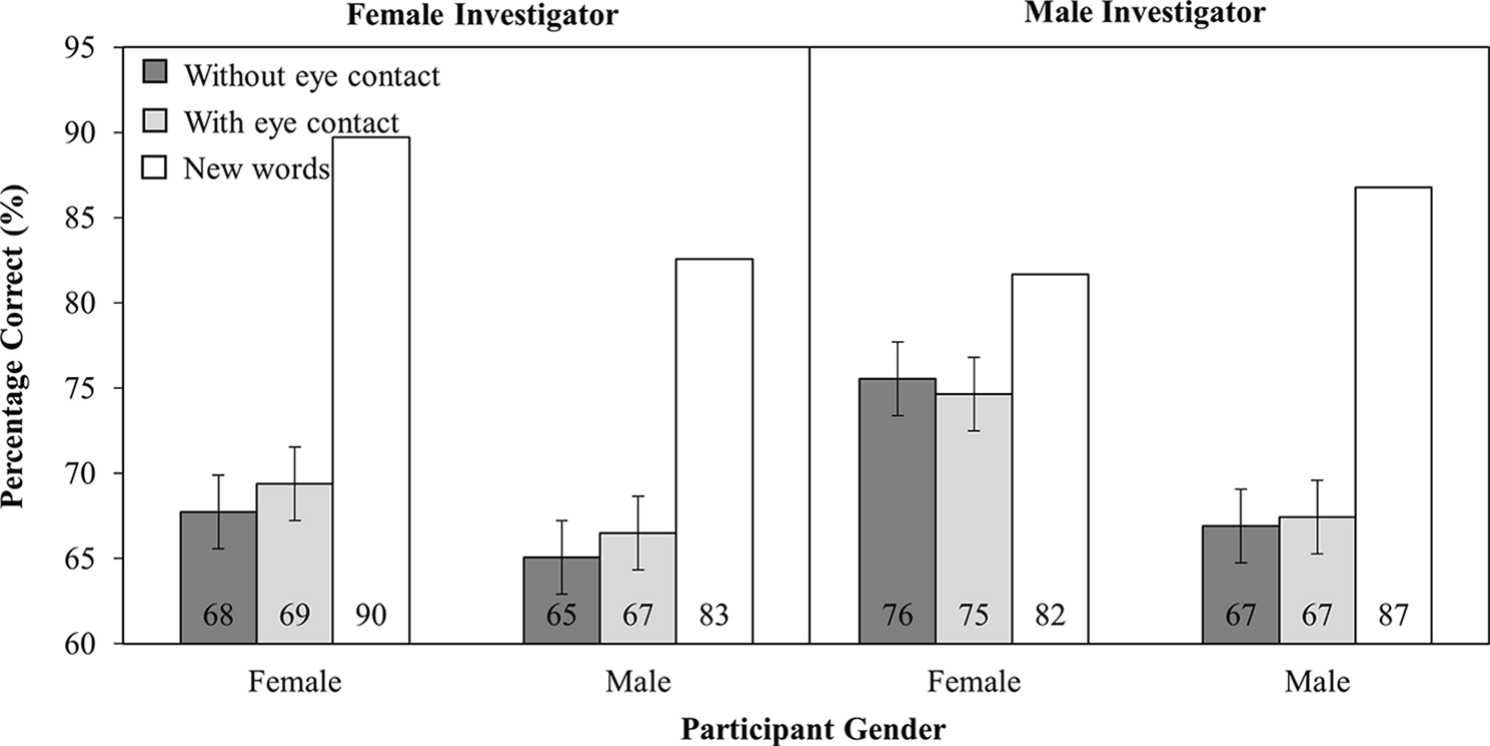
Percentage correct as a function of Investigator Gender (Female versus Male), Participant Gender (Female versus Male), and Investigator gaze (With eye contact versus Without eye contact). Note that new words have been plotted in this figure as a reference point, but were not included in the analysis. Error bars represent the 95% confidence interval as defined by Masson and Loftus (2003).

Analysis of the accuracy data (Figure 9) revealed a main effect of participant gender (*F*_(1,166)_ = 4.43, MSE = 542.79, *p* < 0.05), such that female participants were more accurate (72%) than male participants (66%). No other main effects or interactions were significant (all other *F’s* < 1.4).

#### Comparison Between Live and Videotaped Investigators

To reveal any difference in the memory effects generated by the eye gaze of a live investigator (in Experiments 1 and 2) and a videotaped investigator, a four-way mixed ANOVA was conducted on response accuracy with Investigator gaze (two levels: with eye contact and without eye contact) as the within-participant factor and Investigator presence (two levels: live and videotaped), Investigator gender (two levels: male and female) and Participant gender (two levels: male and female) as between-participant factors.

The analysis of mean RTs revealed that there was a main effect of investigator presence (*F*_(1,328)_ = 12.19, MSE = 199,677.54, *p* < 0.001), such that participants were faster to recognize words that were said by the in-person investigator (987 ms) than the videotaped investigator (1,111 ms). There was also a main effect of investigator gender (*F*_(1,328)_ = 11.77, MSE = 199,677.54, *p* < 0.001), such that participants were faster to recognize words that were said by the male investigator (991 ms) than the female investigator (1,109 ms). There was a marginal interaction between investigator presence and investigator gender (*F*_(1,328)_ = 3.80, MSE = 199,677.54, *p* = 0.05), such that when the investigator appeared over video, participants recognized words that were said by a male investigator (1,018 ms) faster than a female investigator (1,203 ms). However, when the investigator was in-person, participants recognized words that were said by a male investigator (965 ms) as fast as words said by a female investigator (965 ms). No other main effects or interactions were significant (all *F*’s < 2.6).

The analysis of the accuracy data revealed a main effect of investigator presence (*F*_(1,328)=_13.43, MSE = 467.57, *p* < 0.001), such that participants recognized more words said by an in-person investigator (75%) than a videotaped investigator (69%). Critically, there was a three-way interaction between investigator gaze, investigator presence, and participant gender (*F*_(1,328)_ = 15.88, MSE = 44.75, *p* < 0.001). When the investigator was in-person, there was an interaction between investigator gaze and participant gender (*F*_(1,166)_ = 31.38, MSE = 38.85, *p* < 0.001) such that female participants recognized more words that were spoken while the investigator made eye contact (78%) than when they did not (74%; *t*_(83)_ = 4.91, SEM = 0.87, *p* < 0.001). However, male participants recognized fewer words read while the investigator made eye contact (73%) than when they did not (76%; *t*_(83)_ = 3.19, SEM = 1.04, *p* < 0.005). In contrast, the same analysis for the videotaped investigator, presented in Section “Percentage correct” (Experiment 4), yielded no effect of investigator gaze, nor an interaction between investigator gaze and participant gender. These findings indicate that the failure to observe any memory effects in Experiment 4 is because eye gaze from a live investigator is fundamentally different from the eye gaze of a videotaped investigator.

### Discussion

In Experiment 4, there was no evidence that eye gaze displayed over video influenced memory. This was true regardless of the investigator or participant’s gender. This stands in direct contrast with the previous experiments that demonstrated that eye contact from a live investigator improved memory in female participants, and reduced memory in male participants. Taken together, the results demonstrate that people attend to the eye gaze of those they interact with in real life differently than the eye gaze of people depicted in images.

The current data dovetail with a growing body of research suggesting that viewing a live person elicits different neurological (Hietanen et al., 2006; Pönkänen et al., 2011a,b) and behavioral responses (Heath and Luff, 1992) than viewing an image of a person. Indeed, the finding that participants who listened to a live investigator recognized more words, faster than those who listened to a video of the same words, supports this notion. By using images, the present study stripped away the social cues that would typically be present during a live encounter, leaving only the perceptual cues associated with eye gaze. Since the effect of eye gaze was eliminated when presented over video, the implication is that the effects observed previously in Experiments 1–3 were driven by communicative cues associated with eye contact instead of noncommunicative perceptual cues.

## General Discussion

In the present studies, female participants benefited from the investigator’s gaze more than male participants on a subsequent memory test. Specifically, female participants recognized more words in a subsequent memory test when the words were previously associated with eye contact from an investigator than when they were not. This was true regardless of the investigator’s gender (female in Experiments 1 and 3 and male in Experiment 2), and whether the investigator’s gaze was a quick glance (as in Experiments 1 and 2) or a prolonged stare (in Experiment 3). In contrast, male participants showed no benefit of the investigator’s gaze on subsequent memory tests when the investigator’s gaze was held longer (Experiment 3), and actually recognized fewer words on a subsequent memory test when they were associated with brief eye contact from the investigator (Experiments 1 and 2) relative to when they were not. While these findings suggest that eye gaze could provide a useful social cue to pay attention for females, this is not the case for males. Importantly, the data suggest it was something socially communicative about eye gaze (rather than something nonsocially communicative), since eye gaze effects disappeared in the absence of a communicative setting when the investigator was presented over video (Experiment 4).

Our finding that females and not males showed a memory enhancement to gaze contradicts a recent study by Helminen et al. (2016) who showed the opposite. Our methods were very different, such that Helminen et al. read aloud narratives, manipulated eye gaze spontaneously, and tested memory recall. Because of this, it is unclear whether the specific information that listeners recalled in Helminen et al. was spoken while the speaker made eye contact or not because the temporal synchrony between the speaker’s eye contact and spoken information was not controlled. As a result, the data from Helminen et al. (2016) are equivocal as to whether memory effects related to gaze reflect an enhancement from direct gaze or a decline resulting from gaze aversion. Our methods, however, can determine whether memory for words is enhanced or diminished by directly pairing each word with direct or averted gaze, and comparing memory in those conditions to a neutral (no gaze) condition. Our design allowed us to discover that females benefited from eye contact by remembering those words more compared to the control (no gaze) condition, while males showed a reduction in memory for words paired with eye contact compared to the control. Thus, we believe our methods can more accurately depict the how eye gaze modulates memory in males and females.

### Gender Differences in Eye Gaze-Related Memory Effects

These findings converge with a body of literature that suggests males and females attend to social information differently. In comparison to males, females dedicate more attention to social stimuli, such as faces and eyes (Connellan et al., 2000; Lutchmaya et al., 2002) and more easily decode the nonverbal signals exhibited by others (Hall, 1978; Rosenthal et al., 1979; McClure, 2000). More importantly, previous studies have demonstrated that females are more responsive to eye gaze (Bailenson et al., 2001; Bayliss et al., 2005). These data suggest that females are more sensitive to social signals in general and eye gaze in particular, and thus the investigator’s eye contact engaged their attention in the first three experiments (despite being irrelevant to the task).

The general interpretation of the memory effect observed in females is that when eye contact accompanies information, it is interpreted as signaling the intent to communicate information that warrants attention (Duncan, 1972; Niederehe and Duncan, 1974; Kampe et al., 2003; Csibra and Gergely, 2009; Senju and Johnson, 2009a). This is particularly noteworthy since the investigator’s eye contact is actually irrelevant to the task at hand. As such, when people communicate with each other and their message is preceded or accompanied with eye contact, their eye contact serves to highlight the most important parts of their message. It would follow that in the present studies, when the female participant was with the speaker, information spoken with eye contact would be attended more and recognized better than information presented without eye contact.

While female participants noticed, and decoded the social signals associated with the investigator’s gaze with apparent ease, male participants did not. It is possible that male participants were simply insensitive to the investigator’s eye contact and/or did not think the eye contact was an important cue, and dedicated the same amount of attention to words regardless of whether the investigator looked at them or not. However, this explanation seems unlikely given that in male participants, the investigator’s eye contact, if anything, had a negative effect on their performance in Experiments 1 and 2. Moreover, participants were instructed to look at the investigator’s eyes throughout the study, so it seems inconceivable that eye contact with the investigator went unnoticed.

Another intriguing possibility is that the males were sensitive to the investigator’s eye gaze, but were unable to decode which signal the investigator intended to convey since eye contact provides a variety of different social signals. While eye contact may signal to pay attention, as previously discussed, eye contact also signals that one is being monitored (Guerin, 1986; Risko and Kingstone, 2011; Pönkänen et al., 2011b; Freeth et al., 2013; Baltazar et al., 2014; Marschner et al., 2015; Nasiopoulos et al., 2015; Hazem et al., 2017), and facilitates decoding of the emotional and intentional messages of others. Discerning which message is most important and/or appropriate (and requires the most attention) in a given situation may be more challenging for males than females. In the present context, males may have struggled to dissociate which signal embedded in the investigator’s eye contact was most relevant (or if they did make this distinction, they appear not to have acted on it). As such, processing or actively ignoring the investigator’s eye contact may have interfered with the males’ ability to pay attention to information that was being spoken. This idea converges with the finding in previous work that the presence of eyes interfered with performance on a Stroop task (Beattie, 1981; Conty et al., 2010; Nemeth et al., 2013), presumably because processing the eyes required the same cognitive resources (e.g., selective attention) used to perform the task. According to this idea, the brief eye contact provided in Experiments 1 and 2 was too difficult for the males to decode while simultaneously completing another task. As a result, they were unable to dedicate enough attention to the task of attending to what the investigator said when they were looked at, and their performance suffered. However, the less subtle signal provided in Experiment 3 did not alter the previous data pattern, undermining the interpretation that males just need a more salient gaze signal for it to yield a performance benefit.

Another similar, though slightly different, explanation of how a participant’s gender modified whether eye contact had a positive or negative impact on memory comes from a proposal put forward by Conty et al. (2016). The authors proposed that direct gaze first captures one’s attention and then triggers self-referential processing, i.e., a heightened processing of contextual information in relation with the self (Northoff et al., 2006). According to this account, direct gaze can have both positive and negative effects on performance since the tendency to pay attention to the direct gaze of others either facilitates or interferes with performance on a task (e.g., direct gaze may facilitate processing a face, but hinder processing information that is not related to the face). However, once direct gaze has triggered self-referential processing, any information associated with it would be prioritized. Indeed, a large body of research suggests that memory is improved for information processed in relation to oneself (i.e., the self-referential memory effect; Macrae et al., 2002; Northoff et al., 2006; Kim, 2012).

In Experiments 1–3, it is possible that the extent to which the speaker’s eye contact triggered self-referential processing differed between males and females. Females may have processed both the speaker’s eye contact and the self-referential cue it provides simultaneously, or simply processed these two signals more efficiently and sequentially. Thus, any interference (if any was experienced) in hearing what the speaker said, caused by simply processing the speaker’s eye contact, was overridden by the self-referential processing benefit triggered through the speaker’s eye contact. Males on the other hand may notice and process the speaker’s eye contact, but not the self-referential cue it provides. As a result, the speaker’s eye contact only interferes with processing what the speaker says. This could be due to interference caused by processing any self-referential cue in the context of the task (i.e., any self-referential cue could be distracting since it is irrelevant to the task of listening to everything the speaker says), or only self-referential cues conveyed through eye contact. Moving forward, it is still unclear which social signals communicated by the live investigator are being interpreted by participants. The findings are consistent with the idea that eye contact provides a social signal to pay attention, which results in memory benefits for information communicated with eye contact. However, there are many nonverbal social cues conveyed during a live encounter that can influence the way an observer pays attention. In fact, some research might suggest that head movements, rather than eye movements, are more important in eliciting attentional shifts in more natural contexts (Tomasello et al., 1998, 2005; Emery, 2000). Even though we have attributed the effects in Experiments 1, 2, and 3 to a social signal conveyed by eye gaze, it is possible that a different social signal that is also associated with the investigators’ gaze could be driving these effects. Future work could clarify whether it was the investigator’s eye contact or a general social cue that was associated with the eye contact that generated the memory benefits observed in the female participants in the previous studies.

Furthermore, although we have considered the no eye contact condition as the control condition, strictly speaking, it is theoretically possible that our observed memory effects reflect a performance decline without eye contact rather than an enhancement with eye contact. Recall that in previous research, listeners watched a speaker who never made eye contact with any listener in an audience or one who periodically made eye contact with some undefined listeners (Otteson and Otteson, 1980; Sherwood, 1987; Fullwood and Doherty-Sneddon, 2006). Even in instances where a single listener is present (in Experiments 1–3 and in Helminen et al., 2016), it is possible that a listener’s memory was improved for the information spoken while making eye contact with the speaker, and it is also possible that a listener had worse memory for information presented while the speaker avoided eye contact. This reduction in memory could be due to the observer feeling excluded by the speaker (a possibility considered but not addressed by Fullwood and Doherty-Sneddon, 2006) or because the speaker’s gaze directs the observer’s attention elsewhere. That said, the fact that participants in our study performed better in the presence of a real investigator than a videotaped investigator, even in the no eye contact condition, suggests that performance is being enhanced in the eye contact condition rather than diminished in the no contact condition. It remains for future studies, however, to confirm this interpretation by testing the adequacy of our baseline, for instance, comparing it to a situation where the speaker could make eye contact with someone other than the participant.

### Contributions of Live and Non-live Settings to Eye Gaze-Related Memory Effects

By presenting a video of an investigator (Experiment 4) instead of a live investigator (Experiments 1–3), the socially communicative function of eye gaze was removed. A live investigator generates an interactive context in which both the investigator and the participants can convey and observe signals with their eyes (Risko et al., 2012, 2016; Baltazar et al., 2014; Gobel et al., 2015; Jarick and Kingstone, 2015; Myllyneva and Hietanen, 2015; Nasiopoulos et al., 2015; Risko and Kingstone, 2015; Conty et al., 2016; Hietanen, 2016; Hazem et al., 2017). This is not the case when the investigator is presented over video, since the investigator cannot observe any signals that the participants convey through their eye gaze. This notion is also supported by research showing that social centers in the brain are more activated when observing live people than when viewing images of people (Hietanen et al., 2006; Schilbach et al., 2010; Pönkänen et al., 2011a,b; Schilbach, 2015). Viewing a pre-recorded investigator enabled a strong test of whether nonsocial signals embedded in eye gaze could generate memory effects that were previously observed in response to a live investigator’s eye gaze in Experiments 1–3. The results were unequivocal. The memory effects previously observed in response to a live investigator’s eye gaze disappeared. Without a socially communicative context, eye gaze had no effect on memory. This finding provides strong support for the idea that socially communicative signals conveyed through eye gaze influence memory. This also stands in contrast with a noncommunicative explanation of how eye contact could affect memory. The notion above presents a challenge to researchers who have generally assumed that using images enables them to study social aspects of eye gaze present in real life with real people. However, if this assumption is misplaced, and indeed more and more research is suggesting this assumption may be, then there could be broad-reaching implications as researchers have been using images to study the social effects of eye gaze for decades.

### Memory Mechanisms Affected by Eye Contact

While the present work has demonstrated that manipulating a speaker’s eye contact during encoding/consolidation can influence recognition memory for semantic (word) information, it has not explored or manipulated memory for other types of materials (e.g., faces) or different memory processes (e.g., retrieval). An important question related to memory retrieval is whether a speaker’s eye contact affects *recall* as well recognition. While studies from natural settings suggest that viewers generally recall more information when speakers periodically make eye contact than when they do not (Otteson and Otteson, 1980; Sherwood, 1987; Fullwood and Doherty-Sneddon, 2006), this notion remains to be tested in a rigorous paradigm that permits one to assess who is, and is not, receiving eye contact, and what information specifically is being delivered in those moments.

Another interesting question relevant to memory retrieval is whether making eye contact during retrieval will help or hinder this process. Some research suggests that direct gaze during the retrieval process can enhance memory for a face (Hood et al., 2003; Smith et al., 2006). However, this question has yet to be tested in a paradigm that systematically manipulates a speaker’s eye gaze during the retrieval process.

In sum, there are a number of different aspects of memory that could be influenced by eye contact. Future studies could extend the present work by exploring the effects of eye contact on all of the different components of memory mentioned at the outset of this section. They could also, for example, examine whether the effects are eye contact specific, or general to other visual (e.g., pointing) or nonvisual (e.g., verbal) cues.

## Conclusion

The significance of the eyes in human communication has fascinated scientists for centuries. While the present findings only begin to scratch the surface of this broad area of investigation, this work does highlight the importance of systematically examining gender and conducting studies in contexts where eye contact can be communicative. Indeed, in the absence of a communicative context, eye gaze did not modulate recognition performance. This conclusion has tremendous implications for social theories of human communication, memory, and cognition more broadly, as images of the eyes have been used to manipulate and measure social behavior and social neural mechanisms of various cognitive processes. Using real people in future studies will enable the assessment of the social effects of eye gaze in particular, and social signals in general, thereby enhancing our understanding of the cognitive and neural bases of human communication and social interaction.

## Ethics Statement

This study was carried out in accordance with the recommendations of the University of British Columbia’s Research Ethics Board [Towards a More Natural Approach to Attention Research 1-200, certificate #H10-00527, & Research in Cognitive Ethology, #H04-80767], with written and oral informed consent from all subjects. All subjects gave written informed consent in accordance with the Declaration of Helsinki. The protocol was approved by the University of British Columbia’s Research Ethics Board in accordance with the guiding ethical principles of the Tri-Council Policy Statement (TCPS2 2014), the International Conference on Harmonization Good Clinical Practice Guidelines (ICH-GCP) and the requirements of the US Department of Health and Human Services, as set out in the Federal Policy for the Protection of Human Subjects, 45CFR Part 46, sub-part A.

## Author Contributions

SL was the lead investigator for all of the projects reported in this dissertation and was primarily responsible for design conception, data management and analysis, and report composition. AK and MJ acted in a supervisory capacity during project conception and report composition. MZ and CB were involved in data collection, management, and organization.

### Conflict of Interest Statement

The authors declare that the research was conducted in the absence of any commercial or financial relationships that could be construed as a potential conflict of interest.

## Footnotes

^1^www.pstnet.com

^2^We thank an anonymous reviewer for raising the possibility that the same effect might not be obtained with a male investigator and prolonged gaze. Based on the current data indicating that there is no effect of investigator gender or gaze duration, we think a 3-way interaction is unlikely, but we acknowledge that it is possible.

^3^Note that nervous system arousal was not directly measured or analyzed in this study and therefore we cannot be certain that arousal was mediating memory performance. Future studies should incorporate measures of arousal using galvanic skin response and/or pupil dilation to investigate this further.
